# The Role of Inflammatory Pathway Genetic Variation on Maternal Metabolic Phenotypes during Pregnancy

**DOI:** 10.1371/journal.pone.0032958

**Published:** 2012-03-30

**Authors:** Margrit Urbanek, M. Geoffrey Hayes, Hoon Lee, Rachel M. Freathy, Lynn P. Lowe, Christine Ackerman, Nadereh Jafari, Alan R. Dyer, Nancy J. Cox, David B. Dunger, Andrew T. Hattersley, Boyd E. Metzger, William L. Lowe

**Affiliations:** 1 Division of Endocrinology, Metabolism and Molecular Medicine, Northwestern University Feinberg School of Medicine, Chicago, Illinois, United States of America; 2 Genetics of Complex Traits, Peninsula College of Medicine and Dentistry, University of Exeter, Exeter, United Kingdom; 3 Department of Preventive Medicine, Northwestern University Feinberg School of Medicine, Chicago, Illinois, United States of America; 4 Genomics Core, Center for Genetic Medicine, Feinberg School of Medicine, Northwestern University, Chicago, Illinois, United States of America; 5 Department of Human Genetics, University of Chicago, Chicago, Illinois, United States of America; 6 Department of Paediatrics, University of Cambridge, Cambridge, United Kingdom; 7 HAPO Study Cooperative Research Group, Chicago, Illinois, United States of America; South Texas Veterans Health Care System and University Health Science Center San Antonio, United States of America

## Abstract

**Background:**

Since mediators of inflammation are associated with insulin resistance, and the risk of developing diabetes mellitus and gestational diabetes, we hypothesized that genetic variation in members of the inflammatory gene pathway impact glucose levels and related phenotypes in pregnancy. We evaluated this hypothesis by testing for association between genetic variants in 31 inflammatory pathway genes in the Hyperglycemia and Adverse Pregnancy Outcome (HAPO) cohort, a large multiethnic multicenter study designed to address the impact of glycemia less than overt diabetes on pregnancy outcome.

**Results:**

Fasting, 1-hour, and 2-hour glucose, fasting and 1-hour C-peptide, and HbA1c levels were measured in blood samples obtained from HAPO participants during an oral glucose tolerance test at 24-32 weeks gestation. We tested for association between 458 SNPs mapping to 31 genes in the inflammatory pathway and metabolic phenotypes in 3836 European ancestry and 1713 Thai pregnant women. The strongest evidence for association was observed with *TNF alpha* and HbA1c (rs1052248; 0.04% increase per allele C; p-value = 4.4×10^−5^), *RETN* and fasting plasma glucose (rs1423096; 0.7 mg/dl decrease per allele A; p-value = 1.1×10^−4^), *IL8* and 1 hr plasma glucose (rs2886920; 2.6 mg/dl decrease per allele T; p-value = 1.3×10^−4^), *ADIPOR2* and fasting C-peptide (rs2041139; 0.55 ug/L decrease per allele A; p-value = 1.4×10^−4^), *LEPR* and 1-hour C-peptide (rs1171278; 0.62 ug/L decrease per allele T; p-value = 2.4×10^−4^), and *IL6* and 1-hour plasma glucose (rs6954897; −2.29 mg/dl decrease per allele G, p-value = 4.3×10^−4^).

**Conclusions:**

Based on the genes surveyed in this study the inflammatory pathway is unlikely to have a strong impact on maternal metabolic phenotypes in pregnancy although variation in individual members of the pathway (e.g. *RETN*, *IL8*, *ADIPOR2*, *LEPR*, *IL6*, and *TNF alpha*,) may contribute to metabolic phenotypes in pregnant women.

## Introduction

There is a well-established link between obesity, diabetes, and insulin resistance and chronic low levels of inflammation (reviewed in [1]). This link is based on multiple lines of evidence. First, markers of the inflammatory response such as tumor necrosis factor-alpha (TNF-alpha), interleukin-1β (IL-1β), interleukin 6 (IL-6), plasminogen activator inhibitor 1 (PAI1), and C-reactive protein (CRP) are strongly associated with features of the metabolic syndrome in humans [2], [3], [4], [5], [6], [7], [8], [9], [10], [11], [12], [13], [14], [15], [16], [17]. Second, mouse knockout strains of IL-6 [18], TNF-alpha [19], PAI-1 [20], IL-18 [21], IL-1α [22], resistin [23], monocyte chemotactic protein-1 (MCP-1) [24], [25], inter-cellular adhesion molecule 1 (ICAM-1) [26], nitric oxide synthase (iNOS) [27], inhibitor of nuclear factor kappa-B kinase subunit beta (IKK-β) [28], [29], jun N-terminal kinase 1 (JNK1) [30], and suppressor of cytokine signaling 1 (SOCS1) [31] have perturbations in measures of insulin resistance or blood glucose levels. Third, patients with chronic inflammatory conditions such as chronic hepatitis C, rheumatoid arthritis, and inflammatory lung disease are at increased risk for developing diabetes (reviewed in [32], [33]). Lastly, genetic or pharmacological removal or reduction of inflammatory factors can protect humans and high-risk rodent models against insulin resistance independent of obesity, indicating that the inflammatory response plays a primary role in the etiology of insulin resistance and diabetes rather than an effect secondary to obesity.

Many of the effects of the inflammatory response on insulin resistance are mediated via the nuclear factor kappa B (NF-κB) signalling pathway [29], [34], [35], [36]. NF-κB is a transcription factor that regulates multiple cellular processes including cell growth, survival and division, apoptosis, stress, hypoxia, and immune function. Upon stimulation by cytokines, NF-κB is activated by IKK-β and translocated into the nucleus where it regulates transcription of a broad range of genes including the pro-inflammatory cytokines, IL-1, IL-6 and TNF-alpha. Ablation of IKK-β in the liver of mice reduces the induction of IL-1α, IL-1β, IL-6, and RANTES cytokine mRNA in liver by high fat diet [37]. Several studies have demonstrated the effects of disruption of the NF-κB pathway on insulin sensitivity. For example elevated levels of TNF-alpha result in insulin resistance [36], [38], [39], [40], [41]. In hepatocytes, over-expression of a specific inhibitor of NF-κB activation, IκB, prevents an IL-1β-induced decrease in insulin sensitivity [37]. Furthermore, nonsteroid anti-inflammatory drugs, including aspirin and sodium salicylate which suppress IκB proteolysis by inhibiting IKK-β [42], improve insulin resistance and glycemia [43], [44].

During pregnancy the levels of the pro-inflammatory cytokines CRP and TNF-alpha are positively associated with fasting insulin and body mass index (BMI), respectively [45]. In women with gestational diabetes, levels of the anti-inflammatory cytokine, adiponectin, are negatively associated with gestational diabetes while TNF-alpha and its soluble receptors sTNFR-1 and sTNFR-2 are positively associated [46], [47]. Furthermore, Lowe *et al* have shown that among non diabetic pregnant women levels of the pro-inflammatory cytokines PAI-1 and CRP are positively associated with maternal glucose, BMI and C-peptide levels while the levels of the anti-inflammatory cytokine, adiponectin, are negatively correlated with the same phenotypes [48].

To date inflammatory pathway genes have not been shown to play a major role in type 2 diabetes or glucose tolerance. However, most of these results are based on genome wide association studies (GWAS) which do not always provide sufficient genetic coverage of any given gene region. The more exhaustive tagging approach possible in a candidate gene screen may, therefore, detect genetic loci not identified by GWAS.

Since pregnancy is an insulin resistant state, the inflammatory pathway may also be important in glucose regulation during pregnancy and the development of gestational diabetes. To determine whether variation within inflammatory pathway genes impacts glucose regulation during pregnancy and the development of gestational diabetes we tested for association between 31 genes (Table 1) within the inflammatory gene pathway densely tagged with 458 genetic single nucleotide polymorphisms (SNPs, Tables 2 and 3) and measures of maternal metabolism in 5549 mothers of the Hyperglycemia and Adverse Pregnancy Outcome (HAPO) cohort, a large multiethnic, multicenter study designed to address the impact of glycemia less than overt diabetes on pregnancy outcome [49].

**Table 1:** List of genes.

Gene ID	Chr. Location	Gene name
*ADIPOQ*	3q27.3	adiponectin precursor
*ADIPOR1*	1q23.1	adiponectin receptor 1
*ADIPOR2*	12p13.33	adiponectin receptor 2
*CCL2*	17q12	small inducible cytokine A2 precursor
*CRP*	1q32.2	C-reactive protein precursor
*CTLA4*	2q33.2	cytotoxic T-lymphocyte-associated protein 4
*IFIH1*	2q24.2	Interferon-induced helicase C domain-containing protein 1
*IL10*	1q32.1	interleukin 10 precursor
*IL18*	11q23.1	interleukin 18 proprotein
*IL18R1*	2q11.2	interleukin 18 receptor 1 precursor
*IL1RL1*	2q11.2	interleukin 1 receptor-like 1 precursor
*IL1A*	2q13	interleukin 1, alpha proprotein
*IL1B*	2q13	interleukin 1, beta proprotein
*IL1R1*	2q11.2	interleukin 1 receptor, type I precursor
*IL1R2*	2q11.2	interleukin 1 receptor, type 2 precursor
*IL1RL2*	2q11.2	interleukin 1 receptor-like 2 precursor
*IL1RN, IL1RA*	2q13	interleukin 1 receptor antagonist
*IL6*	7p15.3	interleukin 6
*IL6R*	1q21.3	interleukin 6 receptor isoform 2 precursor
*IL8*	4q13.3	interleukin 8 precursor
*IL8RA*	2q35	interleukin 8 receptor alpha
*IL8RB*	2q35	interleukin 8 receptor beta
*KL*	13q13.1	Klotho
*LEP*	7q32.1	leptin precursor
*LEPR*	1p31.2	leptin receptor
*NFKB1*	4q24	nuclear factor kappa-B, subunit 1
*NFKB2*	10q24.32	nuclear factor of kappa light polypeptide gene
*NOD2*	16q12.1	nucleotide-binding oligomerization domain
*PAI1 (SERPINE1)*	7q22.1	plasminogen activator inhibitor-1
*RETN*	19p13.2	Resistin
*TNF*	6p21.33	tumor necrosis factor alpha

**Table 2:** Candidate gene characteristics.

Gene Region	Chr	Size(kb)	htSNPs* submitted	htSNPs* genotypedsuccessfully
*ADIPOQ*	3q27.3	16	11	11
*ADIPOR1*	1q23.1	18	14	13
*ADIPOR2*	12p13.33	98	28	26
*CCL2*	17q12	1.9	0	0
*CRP*	1q23.2	2.3	0	0
*CTLA4*	2q33.2	6.2	0	0
*IFH1*	2q24.2	8.4	0	0
*IL10*	1q32.1	5	18	16
*IL18*	11q23.1	21	12	12
*IL1RL1/IL18R1*	2q11.2	60	30	28
*IL1A/IL1B*	2q13	64	24	19
*IL1R1*	2q11.2	113	19	19
*IL1R2*	2q11.2	37	24	20
*IL1RL2*	2q11.2	52	12	12
*IL1RN/IL1RA*	2q13	33	21	22
*IL6*	7p15.3	4.8	19	18
*IL6R*	1q21.3	63	23	20
*IL8*	4q13.3	3.2	11	9
*IL8RA,B*	2q35	43	16	16
*KL*	13q13.1	50	0	0
*LEP*	7q32.1	16	16	15
*LEPR*	1P31.2	217	75	74
*NFKB1*	4q24	116	43	41
*NFKB2*	10q24.32	7.9	9	9
*NOD2*	16q12.1	11	0	0
*PAI (SERPINE1)*	7q22.1	12	2	0
*RETN*	19p13.2	1.4	12	12
*TNF*	6p21.33	2.8	15	15

* htSNPs = Haplotype tagged SNPs.

**Table 3:** Disease associated SNPs (candSNPs) and coding variant SNPs (cSNPs).

Variant	Gene		Chr. Location	Amino Acids
rs266729	*ADIPOQ*	candSNP [76], [77], [78]	3q27.3	5′ of gene
rs1501299	*ADIPOQ*	candSNP [79], [80], [81], [82]	3q27.3	+276G>T
rs17366743	*ADIPOQ*	cSNP. candSNP [83], [84]	3q27.3	Tyr111His
rs1024610	*CCL2*	candSNP	17q12	5′ of gene
rs991804	*CCL2*	candSNP	17q12	intronic
rs1130864	*CRP*	candSNP [85], [86], [87]	1q23.2	intronic
rs1800947	*CRP*	candSNP [85], [86], [87]	1q32.2	Leu184Leu
rs3087243	*CTLA4*	candSNP	2q33.2	3′ of gene
rs1990760	*IFIH1*	cSNP, candSNP [88], [89], [90], [91], [92], [93]	2q24.2	Ala946Thr
rs16944	*IL1A/IL1B*	candSNP [94]	2q13	intronic
rs6731822	*IL1A/IL1B*	cSNP	2q13	Ile375Val
rs1800871	*IL10*	candSNP [95], [96]	1q32.1	5′ of gene
rs1800896	*IL10*	candSNP [96], [97]	1q32.1	5′ of gene
rs1800872	*IL10*	candSNP [95], [96]	1q32.1	5′ of gene
rs5744256	*IL18*	candSNP [98]	11q23.1	intronic
rs4251961	*IL1RN, IL1RA*	candSNP [99], [100], [101]	2q13	intronic
rs315943	*IL1RN, IL1RA*	candSNP	2q13	3′ of gene
rs1800795	*IL6*	candSNP [102], [103], [104], [105], [106], [107]	7p15.3	intronic
rs952146	*IL6R*	candSNP	1q21.3	5′ of gene
rs4537545	*IL6R*	candSNP [105], [108]	1q21.3	intronic
rs2229238	*IL6R*	candSNP [109]	1q21.3	3′ UTR
rs9536314	*KL*	candSNP and cSNP [110]	13q13.1	Phe352Val
rs12324931	*NOD2*		16q12.1	5′ of gene
rs6465787	*PAI1*	canSNP [111]	7q22.1	5′ of gene
rs2227631	*PAI1*	candSNP [111]	7q22.1	5′ of gene

## Results

To evaluate the impact of the inflammatory gene pathway on glycemia during pregnancy we carried out an association study. We genotyped 508 SNPs mapping to 31 inflammatory genes (Tables 1, 2, 3) in 6218 pregnant women of the HAPO cohort. We tested for association between these genetic markers and six maternal metabolic phenotypes (fasting, 1-hour and 2-hour glucose levels from the OGTT, fasting and 1-hour C-peptide levels from the OGTT, and HbA1C). Each phenotype was tested under a minimally adjusted statistical model and a fully adjusted model.

### Association Testing

The most statistically significant results of the association analyses are shown in Table 4. Two models were analyzed for each outcome. Model I was a minimally adjusted model that included adjustment for field center, ethnicity using the first two principal components, maternal age, parity, gestational age at OGTT, and neonatal gender. Model II was the fully adjusted model, adjusting for all covariates included in model I as well as mean arterial pressure at OGTT, maternal BMI at OGTT, and maternal height at OGTT. While none of our findings reach formal genome wide statistical significance (p-value<1×10^−8^ [50]), several SNPs do show evidence for association (defined as p<5.0×10^−4^) with maternal phenotypes during pregnancy. We arbitrarily selected p<5.0×10^−4^ as nominally significant since it corresponds to a corrected p-value of 0.05 after correction for 100 independent gene regions and the 508 SNPs analyzed in the study fell into ∼100 independent linkage disequilibrium blocks.

**Table 4:** Results of Association Studies.

							Model 1		Model 2	
Phenotype	Gene	SNP	Allele	Cohort	MAF*	N**	Beta	p	Beta	p
1 hourC-peptide (ug/L)	*ADIPOR1*	rs7554506	A	Eur. Anc.	0.355	3830	0.021	***	0.013	***
				Thai	0.086	1713	−0.572	0.0026	−0.065	0.0005
				All	0.272	5543	−0.052	***	−0.068	***
FastingC-peptide(µg/L)	*ADIPOR2*	rs2041139	A	Eur. Anc.	0.005	3800	0.548	0.00014	0.313	0.00850
				Thai	0.043	1710	0.031	***	0.029	***
				All	0.017	5510	0.138	0.02658	0.083	***
1 hour PG(mg/dl)	*IL6*	rs6954897	G	Eur. Anc.	0.428	3833	−2.245	0.00095	−1.814	0.00600
				Thai	0.056	1710	−3.044	***	−3.349	***
				All	0.413	5543	−2.291	0.00043	−1.935	0.00231
1 hour PG(mg/dl)	*IL8*	rs2227306	T	Eur. Anc.	0.439	3833	−2.496	0.00020	−2.231	0.00063
				Thai	0.330	1709	−0.399	***	−0.434	***
				All	0.405	5542	−1.903	0.00086	−1.748	0.00175
1 hour PG(mg/dl)	*IL8*	rs2886920	T	Eur. Anc.	0.438	3833	−2.552	0.00013	−2.297	0.00040
				Thai	0.426	1709	−0.868	***	−0.812	***
				All	0.434	5542	−2.045	0.00026	−1.864	0.00065
1 hour C-peptide(µg/L)	*LEPR*	rs1171278	T	Eur. Anc.	0.179	3833	−0.040	***	−0.056	***
				Thai	0.097	1713	−0.573	0.00086	−0.620	0.00024
				All	0.154	5546	−0.144	***	−0.167	0.03161
1 hour C-peptide(µg/L)	*LEPR*	rs1627238	T	Eur. Anc.	0.179	3835	−0.038	***	−0.049	***
				Thai	0.097	1713	−0.564	0.00104	−0.613	0.00029
				All	0.154	5548	−0.140	***	−0.160	0.03911
FastingplasmaGlucose(mg/dl)	*RETN*	rs1423096	A	Eur. Anc.	0.103	3833	−0.843	0.00078	−0.742	0.00136
				Thai	0.207	1712	−0.554	0.04514	−0.470	***
				All	0.135	5545	−0.721	0.00011	−0.620	0.00039
HbA1c(%)	*TNF*	rs1052248	A	Eur. Anc.	0.264	3648	0.027	0.00427	0.029	0.00140
				Thai	0.361	1567	0.063	0.00747	0.057	0.01296
				All	0.294	5215	0.039	6.99×10^−5^	0.040	4.42×10^−5^
HbA1c(%)	*TNF*	rs11575839	T	Eur. Anc.	0.017	3647	0.004	***	0.010	***
				Thai	0.087	1568	0.121	0.00313	0.122	0.00257
				All	0.039	5215	0.082	0.00052	0.086	0.00025

* MAF – minor allele frequency.

** number of subjects included in the analysis.

*** p-value>0.05.

### Association with glucose levels

**Figure 1:**
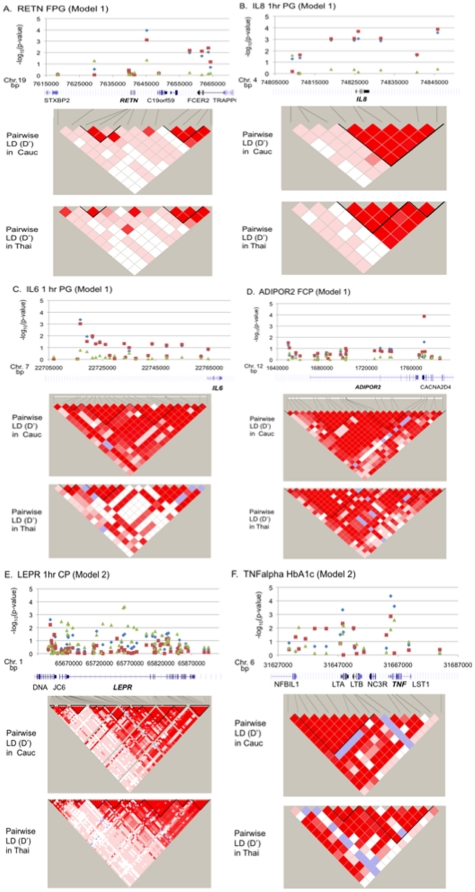
Association results of maternal traits in the HAPO cohort. Blue diamonds correspond to association results for SNPs in the complete cohort (All). The red squares correspond to association results for SNPs in the European ancestry cohort (Eur_Anc). The green triangles correspond to association results for SNPs in the Thai cohort (Thai). The -log_10_p-value for each association test is shown along the y-axis, and the location of each SNP is indicated along the x-axis. The genomic region included in the genetic analysis is illustrated below each panel of association results (based on USC genome browser May 2004 (NCBI35/hg17) build). FPG = fasting plasma glucose levels during OGTT; All n = 5546, European Ancestry n = 3834, and Thai n = 1712. 1-hour PG = 1-hour plasma glucose levels during OGTT; All n = 5543, European Ancestry n = 3833, and Thai n = 1710. 1-hour CP = 1-hour c-peptide during OGTT; All n = 5549, European Ancestry n = 3836, and Thai n = 1713. HbA1c; All n = 5216, European Ancestry n = 3648, and Thai n = 1568.

Multiple genes showed evidence for association with glucose levels. The strongest evidence for association with glucose levels was with variants in the genomic region for resistin (*RETN)*. There was evidence for association with the SNP rs1423096, which maps 5 kb 5′ to *RETN* and fasting plasma glucose (FPG) levels (p-value 1.1×10^−4^ with Model 1) in the complete cohort (Table 4; Figure 1A). Although each cohort by itself demonstrated only moderate evidence for association, the effect size of rs1423096 was very similar in the European ancestry (β = −0.74 to −0.84 mg/dl), Thai (β = −0.47 to −0.55 mg/dl), and combined cohorts (β = −0.62 to −0.72 mg/dl) under each model tested. Thus, these data support a role for rs1423096 on FPG levels in both cohorts and demonstrate that this effect is not sensitive to potential confounding factors.

Two variants (rs2227306, and rs2886920) in the gene encoding interleukin 8 (*IL8*) were associated with 1-hour plasma glucose levels (1-hour PG) at p<5.0×10^−4^ with essentially the same effect size for each SNP (Table 4; Figure 1B). Although the evidence for association is substantially stronger in the European ancestry cohort, the effect, while not statistically significant, is in the same direction in the Thai cohort. These two SNPs are in linkage disequilibrium and, therefore, are likely detecting the same genetic signal.

A SNP mapping 17 kb upstream of interleukin 6, *IL6* (rs6954897) is negatively associated with 1-hour plasma glucose levels with Model 1 in the European ancestry cohort (β = −2.245, p-value = 9.5×10^−4^; Table 4, Figure 1C). Although the effect size was very similar in the Thai population (β = −3.044) the findings did not reach statistical significance due to the reduced MAF (0.056 vs. 0.428 in the European ancestry cohort) of rs6954897 and smaller sample size in the Thai population. However, in the analysis of all samples in model 1, this SNP demonstrated association with 1-hour plasma glucose ((β = −2.291, p-value = 4.3×10^−4^; Table 4, Fig. 1C).

### Association with C-peptide levels

The adiponectin receptor 2 (*ADIPOR2)* SNP, rs2041139, was associated with fasting C-peptide levels in the European ancestry cohort (β = 0.548, p-value = 1.4×10^−4^ in Model 1; Table 4, Figure 1D) but only to a much lesser degree in the Thai cohort (β = 0.031, p-value = not significant). It should be noted that the minor allele frequency (MAF) for rs2041139 is very low in the Thai population (MAF = 0.005); therefore the findings at this locus may not be very robust and need to be interpreted with caution.

Two SNPs within the leptin receptor, *LEPR* (rs1627238 and rs1171278) were associated with 1-hour C-peptide levels in the Thai cohort (Table 4 and Figure 1E; (β = 0.61–0.62, p-value = 2.9×10^−4^ and p-value = 2.4×10^−4^ respectively). Neither marker showed significant evidence for association in the European ancestry cohort. Both markers map within a two kb fragment of intron 2 of *LEPR* and are in strong LD with each other (Figure 1E).

### Association with HbA1C levels

The strongest evidence for association with HbA1C and in the overall study (p-value = 4.42×10^−5^) was observed with rs1052248 in the tumor necrosis factor alpha gene, *TNF*, under the fully adjusted model in the complete cohort (Table 4; Figure 1G). rs1052248 was associated with HbA1c with somewhat stronger effect size in the Thai cohort than the European derived cohort ((β = 0.057 vs. 0.029 in Model 2). One other SNP in *TNF* (rs11575839) also have evidence of association (p<5.0×10^−4^) with HbA1c. In each case the strongest evidence was in the combined cohort with some evidence for association in both populations.

SNPs in several genes showed evidence for association in only one cohort or the effect was in the opposite direction in the Thai and European ancestry cohorts (Table 4). These include rs7554506 in adiponectin receptor 1, *ADIPOR1*, with 1-hour C-peptide levels under the fully adjusted model (Model 2). This association was only observed in the Thai cohort.

## Discussion

In a screen of 31 genes encoding members of the inflammatory pathway, we identified several with evidence for association with metabolic phenotypes in pregnant women. In particular the genes for cytokines including *TNF*, *RETN*, *IL6*, and *IL8* were associated with glucose levels during pregnancy. Two receptors for cytokines (*ADIPOR2* and *LEPR*) were associated with fasting and 1-hour C-peptide levels respectively.

A large number of GWAS have been carried out for type 2 diabetes and intermediate phenotypes such as fasting glucose levels [51], [52], [53], [54], [55], [56], [57], [58], [59], [60], [61], [62], [63], [64], [65]. However, no member of the inflammatory gene pathway was identified among the approximately 40 type 2 diabetes susceptibility loci identified to date nor was there evidence for association with diabetes related phenotypes. These observations can be due to several factors. For one GWAS genotyping arrays on average interrogate 80% of the human genome and therefore may not have exhaustive coverage of specific genic regions of interest. Second, the effect size of the inflammatory genes may be too modest to reach genome-wide significance. Small effect sizes may still be detected in a candidate gene approach due to the reduced level of multiple testing correction that is required. In fact none of our findings reached genome wide significance and would therefore not have been detected in a GWAS. To evaluate the effect of genetic variations in genes in the inflammatory pathway on maternal glycemia during pregnancy with greater sensitivity than possible with current GWAS arrays, we designed a genotyping array with exhaustive coverage containing 458 SNPs in 31 genes belonging to the inflammatory pathway. In addition to tagging SNPs, we also included any known coding variants (cSNPs) mapping to the candidate genes and any SNPs previously identified as candidate SNPs (candSNPs) within the candidate genes (highlighted in Table 3).

While the loci with strongest evidence for association with metabolic traits in our study have not been reported in published GWAS, it is possible that they are associated at significance levels below the traditional cut-off reported in these studies. In fact multiple markers in both *ADIPOR1* and *IL8* do show evidence (p-value<0.01) for association with metabolic measures in the MAGIC consortium (Meta-Analyses of Glucose and Insulin-related traits Consortium) meta analysis results [61], [66]. Notably there was no evidence for association with markers mapping to *LEPR*, *TNF*, *IL6*, *ADOPOR2*, or *RETN* even in the same SNP that was associated in our study as was the case for *LEPR*, *TNF*, *IL6*, and *RETN*. Markers within *IL10* were nominally associated with glucose levels (p-value ∼0.02) in the MAGIC consortium. One potential explanation for these observations is that some of our findings are unique to pregnancy (*LEPR*, *TNF*, *IL6*, and *RETN*) while others are also observed in the non-pregnant population. This would not be surprising since during pregnancy there is an increase of the inflammatory response due to both the general insulin resistant state of pregnancy and due to placentally derived factors.

Replication of genetic association results is critical for the validation of a potential susceptibility locus. In this study we analyze two large cohorts of European and Asian ancestry. Not only does this allow us to replicate our results from one cohort in the other, it also provides evidence for the generalizability of our findings to global populations. As is the case for most other common diseases, genetic analysis of the inflammatory gene pathway in metabolic phenotypes has been predominantly limited to populations of European ancestry. In our study 16 out of 17 of our strongest signals show evidence for association that is consistent in both populations (i.e. in the same direction).

The genetic effect of some of the inflammatory genes highlighted in this study has been previously studied. Variants within *IL18*, *IL1RN*, *IL6R*, and *PAI1* showed no evidence for association with type 2 diabetes in a large meta- analysis [67]. In an in-depth analysis Boraska *et al* tested for association between type 2 diabetes and nine tagging SNPs mapping to the TNF alpha/LTA gene region in 1520 cases and 2570 controls but found no evidence for association [68]. Hivert *et al* tested for association between 21 tagging SNPs in *RETN* and diabetes related traits in 2531 members of the Framingham Offspring Study cohort [69]. They found significant evidence for association 3′ to RETN with both resistin and fasting glucose levels. Although, they tested rs1423096 which is the variant we found to be associated with fasting glucose levels in this study (Table 4 and Figure 1A), the strongest evidence for association with glucose in the Framingham Offspring Study was with rs10401670 which maps 3,626 bp downstream of rs1423096. We detect no evidence of association between rs10401670 and any phenotypes tested in the HAPO cohort. Hivert *et al* carried out a similar analysis for 22 tagging SNPs mapping to the adiponectin gene (*ADIPOQ*). They found evidence for association with the C allele of nonsynonymous SNP in exon 3 of the gene (rs17366743, Y111H) and diabetes and fasting plasma glucose levels. While we do not observe statistically significant evidence for association between this variant and glucose levels in our study, there is a trend for association with the C-allele of rs17366743 with 2-hour plasma glucose in European ancestry pregnant women (p-value = 0.081). It should be noted that, while T2D and elevated glucose levels during pregnancy are related phenotypes, they are not identical and may therefore have different susceptibility loci.

To date most of genetic studies of the role of inflammatory genes in metabolic phenotypes have been limited to one or two functional variants per gene and small sample sizes (n<1000). The impact of genetic variation within the leptin receptor (*LEPR*) on metabolic phenotypes has been intensively studied but the findings remain ambiguous. For instance, both alleles of the coding variant Gln223Arg (rs1137101) of the *LEPR* have shown evidence for association with increased BMI. While we did see nominal evidence for association between rs1137101 and HBA1C levels in the Thai population and 1-hour C-peptide levels in the Caucasian cohort (data not shown), these results did not reach significance as defined in our study. To date no major studies have been carried out to systematically assess the impact of this pathway on maternal metabolic phenotypes during pregnancy.

Past studies have shown clear evidence for association between protein levels of various members of the inflammatory pathway and metabolic phenotypes including insulin resistance, type 2 diabetes, and obesity. However these studies cannot provide evidence for the directionality of the effect – in other words does insulin resistance lead to perturbations of the inflammatory pathway or vice versa. Genetic studies such as the one presented here can address this question. Our data support the hypothesis that variation within the inflammatory pathway itself impacts metabolic phenotypes during pregnancy rather than the reverse. Although several of the findings presented here (association between *TNF alpha* and HbA1c, *IL8* and 1-hour plasma glucose, *IL6* and 1-hour plasma glucose, and *RETN* and fasting plasma glucose) show clear evidence for association in independent populations and therefore provide evidence of replication within this cohort, further studies are needed to determine whether the findings presented here are limited to pregnancy or can be expanded to other phenotypic groups.

### Conclusions

Based on the genes surveyed in this study, we observed significant evidence for association between members of the inflammatory gene pathway and several metabolic measures during pregnancy. Specifically, cytokines were associated with fasting glucose (*RETN)*, 1-hour plasma glucose levels during the OGTT (*IL6, IL8)* and HbA1C levels (*IL10, TNF*) while cytokine receptors were associated with fasting (*ADIPOR2*) or 1-hour C-peptide (*LEPR)* levels. Thus, this study suggests that while some members of the inflammatory gene pathway are associated with metabolic phenotypes in pregnant women, the inflammatory pathway is unlikely to have a strong impact on maternal metabolism.

## Methods

HAPO was an international, multi-center epidemiologic study conducted at 15 centers in 9 countries. The HAPO study was approved by the local institutional review board at each center. The centers contributing subjects studied in this work are Bangkok (Rajavithi Hospital, Ministry of Public Health), Manchester (Health and Social Care Trust Research Ethics Committee), Belfast (Health and Social Care Trust Research Ethics Committee), Brisbane (Mater Medical Research Institute), and Newcastle (Hunter Area Research Ethics Committee). All participants gave written informed consent. An external Data Monitoring Committee provided oversight. Study methods have been published [70], [71], [72]. A brief overview is presented here.

### Participants

All pregnant women at each field center were eligible to participate unless they had one or more exclusion criteria [70]. Gestational age and the expected date of delivery were determined as previously described [70].

### Oral glucose tolerance test (OGTT)

All participants underwent a standard 75-gram OGTT between 24 and 32 weeks gestation and as close to 28 weeks as possible. Plasma glucose samples were collected at fasting and one- and two-hours after the glucose load. Samples for serum C-peptide were collected at fasting and one-hour time points (Table 5).

**Table 5:** Baseline characteristics of study participants.

	Belfast, UK	Manchester, UK	Brisbane, Australia	Newcastle, Australia	Bangkok, Thailand
Pregnant women	1288	1098	959	490	1695
Gestational age at OGTT (weeks)	29.0±1.2	28.4±1.0	28.1±1.2	28.0±1.5	28.1±1.8
Age at OGTT (years)	29.8±5.5	30.8±5.6	29.2±5.3	29.5±5.5	27.9±5.5
BMI at OGTT (kg/m^2^)	28.4±4.9	29.0±5.5	29.0±5.7	29.7±6.1	25.7±3.7
Mean arterial pressure at OGTT (mm Hg)	83.6±7.9	83.5±8.0	83.8±7.6	82.7±8.1	80.3±7.7
Fasting Plasma Glucose (mmol/l)	4.6±0.4	4.6±0.4	4.4±0.4	4.5±0.4	4.5±0.4
1-hour plasma glucose (mmol/L)	7.5±1.7	7.5±1.8	7.4±1.5	7.3±1.7	8.3±1.7
2-hour plasma glucose (mmol/L)	6.1±1.2	6.0±1.4	6.2±1.2	6.2±1.3	6.7±1.4
Fasting C-peptide (ug/L)	2.1±0.9	2.1±0.9	1.6±0.8	2.1±0.9	1.7±0.8
1-hour C-peptide (ug/L)	10.1±3.1	9.8±3.0	7.7±2.5	9.5±2.8	10.0±3.0
HbA1c (%)	4.8±0.4	4.9±0.3	4.8±0.4	4.8±0.4	4.5±0.6

### Anthropometric measurements

Height, weight, and blood pressure were measured at the OGTT visit using standardized procedures and calibrated equipment. Personal and demographic data were collected using standardized questionnaires. Race/ethnicity was self-identified (Table 5).

### Unblinding

In order to make timely clinical decisions, aliquots of fasting and 2-hour OGTT or random plasma glucose (RPG) samples were analyzed enzymatically at field center laboratories (12). Values were unblinded if FPG exceeded 5.8 mg/dl (105 mg/dL)), if 2-hour PG exceeded 11.1 mg/dl (200 mg/dL)), if RPG was ≥8.9 mg/dl (160 mg/dL) or if any PG value was less than 2.5 mg/dl (45 mg/dL). Otherwise, women, caregivers, and HAPO Study staff (except for laboratory personnel) remained blinded to glucose values. To avoid center-to-center analytical variation, aliquots of the OGTT glucose and C-peptide samples were analyzed at the HAPO Central Laboratory [73]. Those results were used for this report. A “Vitros 750” analyzer was used for glucose analysis and serum C-peptide was assayed on an Autodelfia instrument [73]. The technical errors for the glucose and C-peptide measurements were 2.0 and 4.2%, respectively.

### DNA sample collection, preparation, and genotyping

A blood sample for DNA extraction was collected into an EDTA tube at 2-hours during the OGTT on women who specifically consented to this. Samples were frozen and shipped to Chicago. DNA was prepared using the automated Autopure LS from Qiagen. For this study we selected the two populations (Caucasians of European ancestry and Thai women from Bangok) with the largest sample size and relatively homogenous ethnic ancestry.

#### SNP selection

Single nucleotide polymorphisms (SNPs) included in this study belong to three categories: (i) anonymous candidate gene SNPs or haplotype tagging SNPs (htSNPs which map to genes belonging to the inflammatory pathway (Table 2), (ii) coding SNPs (cSNPs) that map to candidate genes (Table 3), and (iii) candidate SNPs (candSNPs) which have demonstrated evidence for association with inflammatory phenotypes in previous studies (Table 3). Anonymous candidate gene SNPs mapping to candidate genes or htSNPs were selected using the HAPMAP Tagger function (http://www.hapmap.org/cgi-perl/gbrowse/hapmap_B35/) to tag the entire genomic segment encompassing a given candidate gene in Caucasians (i.e. the CEPH trios genotyped by HAPMAP), Asian (Chinese and Japanese HAPMAP samples), and African (Yoruban HAPMAP trios) at an r^2^ of ≥0.8. A candidate gene region was identified as the coding region of the gene plus 20 kb upstream and 15 kb downstream of the gene. The SNPs consisted of 454 candidate gene tagging SNPs (htSNPs, Table 2), 54 candidate and/or coding SNPs (candSNPs, cSNPs, Table 5). In addition we genotyped 50 previously identified Ancestry Informative Markers (AIMs). At the time of SNP selection neither results from GWA studies in T2D and related phenotypes nor panels of SNP based AIMs were available.

#### Genotyping

SNPs were genotyped using the Illumina Goldengate Assay on 32 sample universal BeadChip and iScan system (Illumina, San Diego CA) in a 1536 SNP bundle according to the manufacturers' recommendations. Each 96 well plate consisted of 93 study samples and one CEPH (Centre d'Etude du Polymorphisme Humain) trio that served as a genotyping control. Two of the CEPH DNAs were randomly positioned on each tray while one DNA was plated in a fixed position on each tray allowing for unambiguous identification of each DNA tray and its orientation. Initial genotyping was carried out using the manufacturer supplied genotype clusters. Genotypes based on these cluster definition were used to identify poor quality DNAs. We defined poor quality DNAs as DNAs with p10GC<0.40, p50GC<0.60 or call rates less than 0.65. The poor quality DNAs were removed from the analysis as were two 96 well trays of DNAs with high rates of gender and replication errors. Samples were re-clustered and genotype clusters for each SNP were examined visually and cluster boundaries were adjusted as necessary. Finally, DNAs were re-genotyped using new cluster definitions. After re-clustering individuals were excluded due to: (i) phenotypic discrepancies including improper unblinding, nonsensical data, incorrect ethnicity, or “bad “IDs, (ii) missing genotypes for >6% of SNPs, or (iii) mom-baby pairs with Mendelian errors for >5% of SNPs.

Of the 508 inflammatory gene SNPs that were submitted for genotyping, 32 markers were dropped due to poor resolution of genotyping clusters. A further 6 SNPs were dropped due to high proportion of genotyping failures (>1% of DNA samples), and 12 SNPs were dropped due to low minor allele frequency (MAF<0.01). Thus 458 SNPs fulfilled our quality control criteria. The average call rate of the SNPs used in the association analyses was 99.9% and average minor allele frequency (MAF) was 0.28. The replication error rate in the Centre D'Etude du Polymorphisme Humain (CEPH) trio was <0.01%. Of the 6218 unique DNA samples submitted for genotyping 5549 subjects (1713 Thai and 3836 Northern European ancestry) remained after data cleaning. 60 DNA samples failed Illumina genotyping quality scores, 441 samples had missing genotypes at greater than 6% of SNPs and 168 samples were dropped due to excessive Mendelian errors (>5%), or due to phenotypic discrepancies including improper unblinding, nonsensical data, incorrect ethnicity, or incorrect sample identifiers. Average call rate for the 5549 samples used in the genetic analyses was >99%.

### Genetic Analysis

Principal Component Analysis (PCA): We used SMARTPCA in the EIGENSOFT software package to calculate principal components for each subject using 145 AIMS. The AIMs consisted of 50 previously identified AIMs and 95 independent htSNPs from this study. These independent htSNPs were chosen by selecting one SNP from each of the genomic regions interrogated in the complete candidate gene 1536 SNP panel. These genomic regions possessed the greatest sum of interpopulation allele frequency differences in the three HapMap Phase I populations, and were not in linkage disequilibrium (r^2^<0.3) with any SNP showing evidence for association (p<0.01) with the six primary phenotypes investigated (see below). This set of 145 SNPs was sufficient to differentiate between the Thai and Caucasians, although choosing a second SNP per genomic region that met the first two conditions above and was not in linkage disequilibrium (r^2^<0.3) with the first SNP selected from the region improved performance. The principal components derived from these analyses were used as covariates to adjust for ethnic variation in the association tests.

#### Association Testing

We tested for association between SNPs and six maternal phenotypes in the complete cohort while adjusting for field center and ethnicity and in Thai and Caucasian cohorts separately. Phenotypes tested were glucose levels (fasting, 1-hour and 2-hour), C-peptide levels (fasting and 1-hour), and HbA1c levels. All phenotypic measurements were obtained at the OGTT. Associations were assessed through linear regressions, with single outcomes under an additive model using PLINK, version 1.05 [74], integrated into the BC/SNPmax database, version 2.5.9 (Biocomputing Platforms Ltd., Espoo, Finland). Natural log (ln) transformation was used to achieve normal distribution for C-peptide levels. Mendelian error checking of genotypes was performed with PedCheck, version 1.00 [75] through BC/SNPmax.

Two models were analyzed for each outcome. Model I was a minimally adjusted model that included adjustment for field center, ethnicity using the first two principal components and four variables that are major covariates: maternal age, parity, gestational age at OGTT, and neonatal gender. Model II was the fully adjusted model, adjusting for all covariates included in model I as well as MAP at OGTT, maternal BMI at OGTT, and maternal height at OGTT.
